# Correlation of transcutaneous and serum bilirubin levels in late preterm and term neonates at a tertiary care center in south India

**DOI:** 10.12688/f1000research.162608.2

**Published:** 2025-09-02

**Authors:** Doreswamy Chandranaik, Shahla Zakir, Laxmi Kamath, Nutan Kamath, Suchetha S Rao

**Affiliations:** 1 Department of Paediatrics, Kasturba Medical College Mangalore, Manipal Academy of Higher Education, Manipal, India

**Keywords:** Transcutaneous bilirubinometer; bilirubin; correlation analysis; phototherapy; Bland Altman analysis; noninvasive; Neonatal jaundice

## Abstract

**Background:**

Neonatal jaundice is one of the most prevalent condition during first week of life causing morbidity and even mortality in few, especially in low – middle income countries. Although visual inspection for jaundice has been a time tested method, serum bilirubin is the gold standard investigation of choice. Due to this newborn receive many heel or vein pricks for testing, hence transcutaneous bilirubinometer can be a helpful non-invasive tool for diagnosing jaundice requiring phototherapy.

**Methods:**

This prospective study was carried out in a tertiary care hospital in Mangalore, Karnataka to compare a non invasive method of detecting bilirubin levels and serum bilirubin levels. Performance of a transcutaneous bilirubinometer Dräger Jaundice Meter JM-105 was assessed against routine venous serum bilirubin testing before phototherapy during neonatal care in the first two weeks of life. Results were derived by analysing the correlation coefficient between two methods and direct agreement was analysed using Bland Altman analysis.

**Results:**

Total of 271 neonates (>35 weeks) were included in the study. Transcutaneous bilirubinometry and serum bilirubin values were done on all of them in the first week of life. Correlation analysis showed significant relationship with a Pearson correlation coefficient of 0.629. Values of transcutaneous bilirubinometer showed excellent agreement with venous serum bilirubin concentration in Bland Altman analysis.

**Conclusions:**

The transcutaneous bilirubinometer is a reliable tool to screen neonates and identify those needing phototherapy there by reducing invasive blood sampling.

## Introduction

Neonatal hyperbilirubinemia is a prevalent condition in newborns, marked by the appearance of jaundice within the first week after birth. It affects approximately 60% of term neonates and up to 80% of preterm neonates. This condition occurs due to the accumulation of unconjugated bilirubin, a lipid-soluble pigment, in the skin and mucous membranes, leading to a yellowish discolouration. ^1^ Neonatal hyperbilirubinemia, while generally benign, is commonly seen postnatally in newborns. However, premature neonates and certain high-risk groups are more susceptible to severe forms, which, if not managed, can progress to complications like kernicterus. ^2^ Neonatal jaundice is clinically identified by a yellowish discolouration of the sclera, skin, and mucous membranes resulting from increased bilirubin levels in the bloodstream. The condition is categorised into two types: Unconjugated Hyperbilirubinemia and Conjugated Hyperbilirubinemia. ^3^ Phototherapy and exchange transfusion are the primary interventions for the prevention and management of bilirubin encephalopathy.

The primary methods for assessing bilirubin levels in newborns include visual inspection, transcutaneous bilirubinometry, and measurement of total serum bilirubin. Visual assessment is simple using Kramer’s rule but has notable limitations, as it is highly subjective; factors such as the physician's experience, the baby's skin colour, clothing, and lighting conditions can all influence the accuracy of visual estimation. Transcutaneous bilirubinometry provides a non-invasive alternative, whereas total serum bilirubin measurement continues to be the gold standard for accurate assessment. Requiring a blood sample for confirmation, especially in high-risk cases. Transcutaneous bilirubin (TCB) assessment uses a handheld electronic device to measure bilirubin levels non-invasively on the skin's surface, providing a painless and convenient method for screening jaundice in term and near-term neonates. It is increasingly accepted in clinical settings due to its simplicity and effectiveness. The device, Transcutaneous Jaundice Detector (Drager Model MBJ20). utilises optical spectroscopy by emitting light into the skin and analysing the reflected wavelengths to estimate total serum bilirubin levels. This method offers a reliable alternative for early jaundice detection without requiring blood draws. ^4^

The TCB works by correlating the amount of light absorbed by bilirubin with its concentration in the skin, providing an estimate of bilirubin levels. The National Institute for Health and Care Excellence (NICE) guidelines advise against using transcutaneous bilirubin (TCB) measurements within the first day of life or for neonates born before 35 weeks of gestation. Despite these limitations, TCB is a non-invasive screening method used to determine the need for phototherapy. Potentially reducing infection risks. In contrast, total serum bilirubin (TSB) measurement involves drawing a blood sample. The blood sample report is plotted on a nomogram, which is hour-specific to assess the neonatal hyperbilirubinemia risk. ^2^

For monitoring bilirubin levels before and after phototherapy in both term and preterm neonates, total serum bilirubin (TSB) is the most reliable standard. However, Obtaining blood samples via heel stick or venipuncture is not only painful and time-intensive but also elevates the risk of local and systemic infections, particularly in preterm neonates. ^4^

Although TCB is an established screening method worldwide, its reliability may vary depending on ethnicity, skin pigmentation, hydration status and health system context. ^5, 6^ In India, available studies are relatively few ^7, 8^ and most are from limited sample sizes. This highlights a research gap, as findings from Western and East Asian populations ^9– 13^ cannot always be extrapolated to Indian neonates. Moreover, darker skin pigmentation may affect optical bilirubin detection, ^5^ underlining the need for region-specific validation. Against this backdrop, our study provides novel data by evaluating TcB in late preterm and term neonates in South India, with a larger sample size and additional analysis of correlation with hours of life and gestational age. This strengthens the evidence base for TcB adoption in resource-limited neonatal units especially southern India.

## Methods

This prospective study was carried out at a tertiary care NICU in a tier 2 city of south India following approval from the Institutional Ethics Committee, Kasturba medical college, Mangalore (Reg No. ECR/541/Inst/KA/2014/RR-20) with approval No IEC KMC MLR 08/2024/543 approved on 21/08/2024. The study is done as per STROBE guidelines for cross-sectional observational study. We adhered to all ethical parameters as per Declaration of Helsinki. The primary objective was to examine the correlation between transcutaneous bilirubin (TCB) and total serum bilirubin (TSB) levels in neonates with jaundice who required phototherapy.

The study included 271 neonates admitted to the NICU between August 2024 and December 2024. Both term and preterm neonates (>35 weeks) with clinical jaundice were included, while neonates with major congenital anomalies, skin conditions affecting the forehead or sternum, or those who had already received phototherapy or undergone exchange transfusions were excluded.

For each neonate, demographic details, antenatal history, maternal complications, feeding patterns, and clinical examination findings were documented using a structured pro forma. Bilirubin levels were assessed using two methods:

1. Transcutaneous Bilirubin (TCB): Measurements were taken using a Transcutaneous Jaundice Detector (Model MBJ20). Three readings were taken over the mid-sternum by the duty doctor, and their average was recorded in mg/dL.
2. Total Serum Bilirubin (TSB): Venous blood samples were collected, and TSB levels were measured using standard laboratory methods.

Each neonate underwent TCB and TSB levels at the same time, and these values were compared for correlation analysis. Data was systematically recorded in an Excel sheet.

### Sample size

To detect a mean difference of 0.23 mg/dL between TSB and TCB measurements with a statistical power of 80% and a significance level of p = 0.05, the required sample size was calculated as 271. This value considers the variability in measurements (σ=1.75) and the critical value for a 95% confidence interval (Zα/2=1.96} = 1.96). A design effect 1.17 was incorporated to account for potential clustering or variability across different population subgroups. This adjustment ensures the study is adequately powered to detect clinically significant differences while maintaining the robustness of the results. This calculation aligns with findings from a previous study, which reported a mean TSB of 8.54 mg/dL and highlighted a standard deviation of 1.75 mg/dL in the average difference between TSB and TCB measurements, with negligible variation by gestational age or ethnicity. These findings provide a reliable basis for estimating the sample size required to achieve statistical validity in detecting the specified mean difference. ^14^

### Statistical analysis

Categorical variables were expressed as frequencies and percentages (%), while continuous variables were summarised as means ± standard deviations (SD) and medians with interquartile ranges (25th–75th percentiles). To evaluate the association between TCB and TSB levels, the Pearson correlation coefficient was used, accounting for factors such as hours of life and gestational age. Bland-Altman analysis was performed to assess the agreement limits between TCB and TSB. Data entry was completed using Microsoft Excel, and statistical analyses were carried out using SPSS software (version 29). A p-value below 0.05 was considered statistically significant.

## Results

A total of 271 neonates requiring phototherapy were included in the study to assess the correlation between transcutaneous bilirubin (TCB) and serum bilirubin (TSB) levels. Among the subjects, 140 (51.66%) were male neonates, and 131 (48.34%) were female neonates.

Table 1 provides a summary of the baseline characteristics of the study participants.

**Table 1.** Description of Baseline characteristics.

Baseline Characteristics	Value (n=271)
Mean gestational age (weeks)	38.34 ± 1.43
Mean hours of life at phototherapy (hours)	54.29 ± 27.2
Mean birth weight (kilograms)	2.91 ± 0.44
Male: Female	140:131 (1.06:1)
NVD (normal vaginal delivery)	49.07% (133)
LSCS (lower segment cesarean section)	50.9% (138)

**Figure 1.**
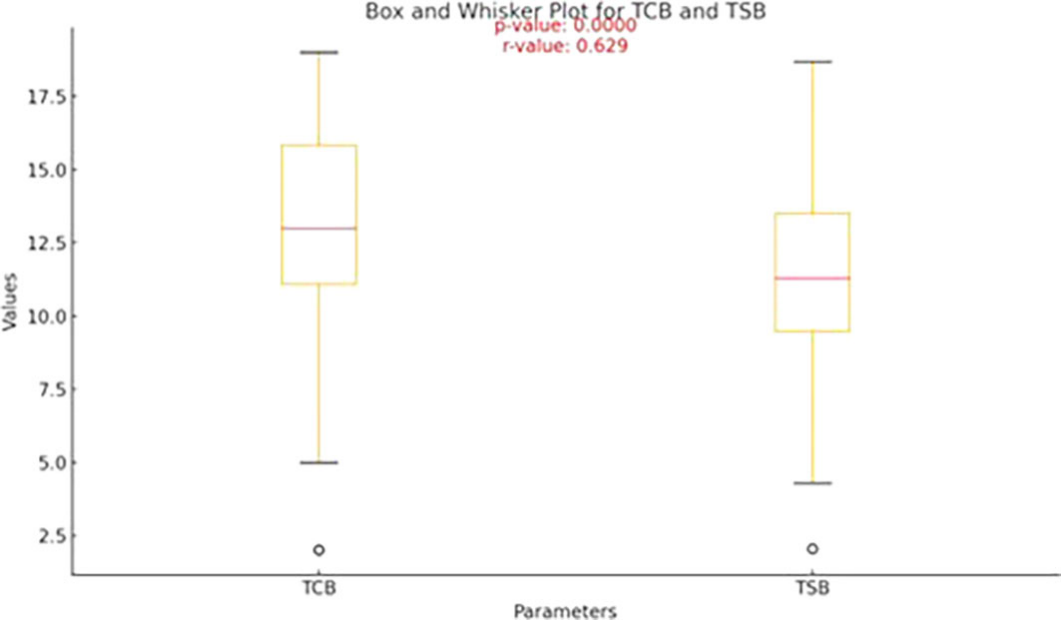
Box-and-whisker plot showing the median values of TCB and TSB.

Table 2 outlines the descriptive statistics for transcutaneous bilirubin (TCB) and serum bilirubin (TSB) levels ( Figure 1).

**Table 2.** Descriptive statistics of transcutaneous bilirubin and Serum Bilirubin.

Variable	Mean ± SD	Median (25th–75th percentile)	Range
Transcutaneous bilirubin (mg/dL)	13.2 ± 3.14	13 (11.1–15.85)	2–19
Serum bilirubin (mg/dL)	11.4 ± 2.97	11.3 (9.5–13.5)	2.04–18.7

**Figure 2.**
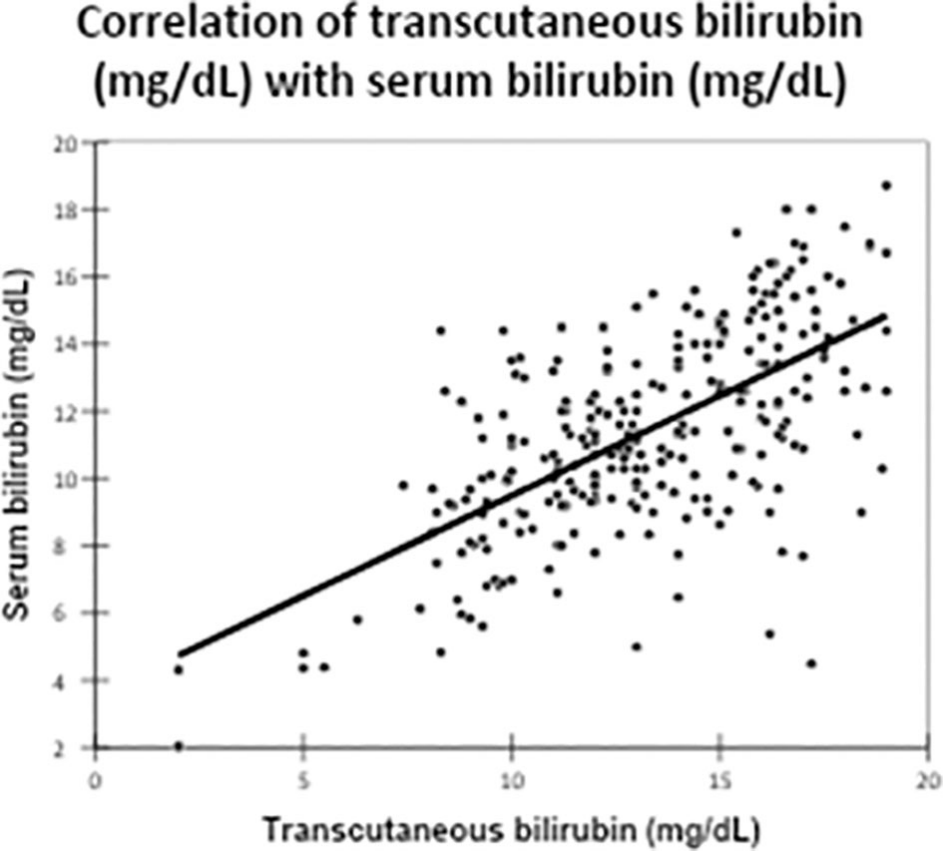
Correlation of transcutaneous bilirubin (mg/dL) with serum bilirubin (mg/dL).

There was a significant positive correlation between TCB and TSB levels, with a Pearson correlation coefficient of 0.629 ( Table 3, Figure 2). This correlation was statistically significant, with a p-value of <0.0001.

**Table 3.** Correlation of Transcutaneous bilirubin with serum bilirubin: Pearson correlation coefficient.

Variables	Serum bilirubin (mg/dL)	Transcutaneous bilirubin (mg/dL)
Pearson Correlation coefficient:		0.629
P value:		<0.0001

The correlation of TCB and TSB levels with hours of life and gestational age is summarised in Table 4.

**Table 4.** Correlation of transcutaneous bilirubin and serum bilirubin with hours of life, gestational age.

Variables	Transcutaneous bilirubin (mg/dL)	Serum bilirubin (mg/dL)
**Hours of life**		
Correlation coefficient	0.620	0.385
P value	<0.0001	<0.0001
**Gestational age (weeks)**		
Correlation coefficient	-0.081	-0.008
P value	0.186	0.894

**Figure 3.**
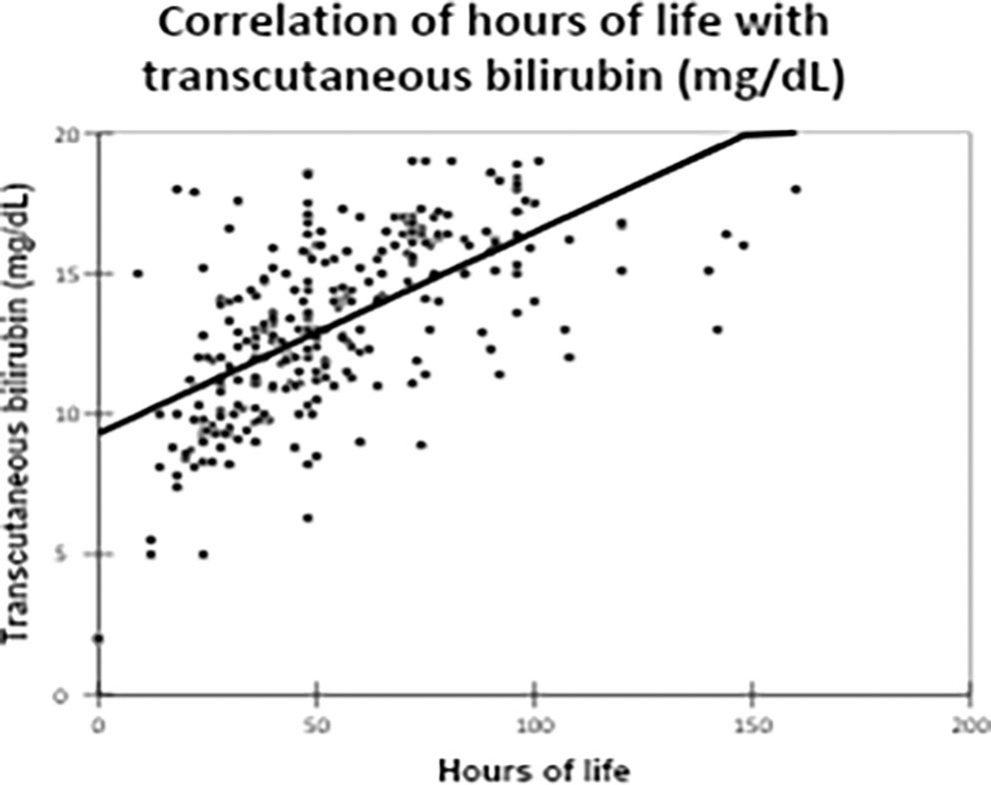
Correlation of hours of life with transcutaneous bilirubin (mg/dL). Agreement Between TCB and TSB.

TCB levels demonstrated a strong positive correlation with hours of life (correlation coefficient: 0.620, p-value: <0.0001), indicating that TCB is beneficial during the first five days of life Figure 3.

**Figure 4.**
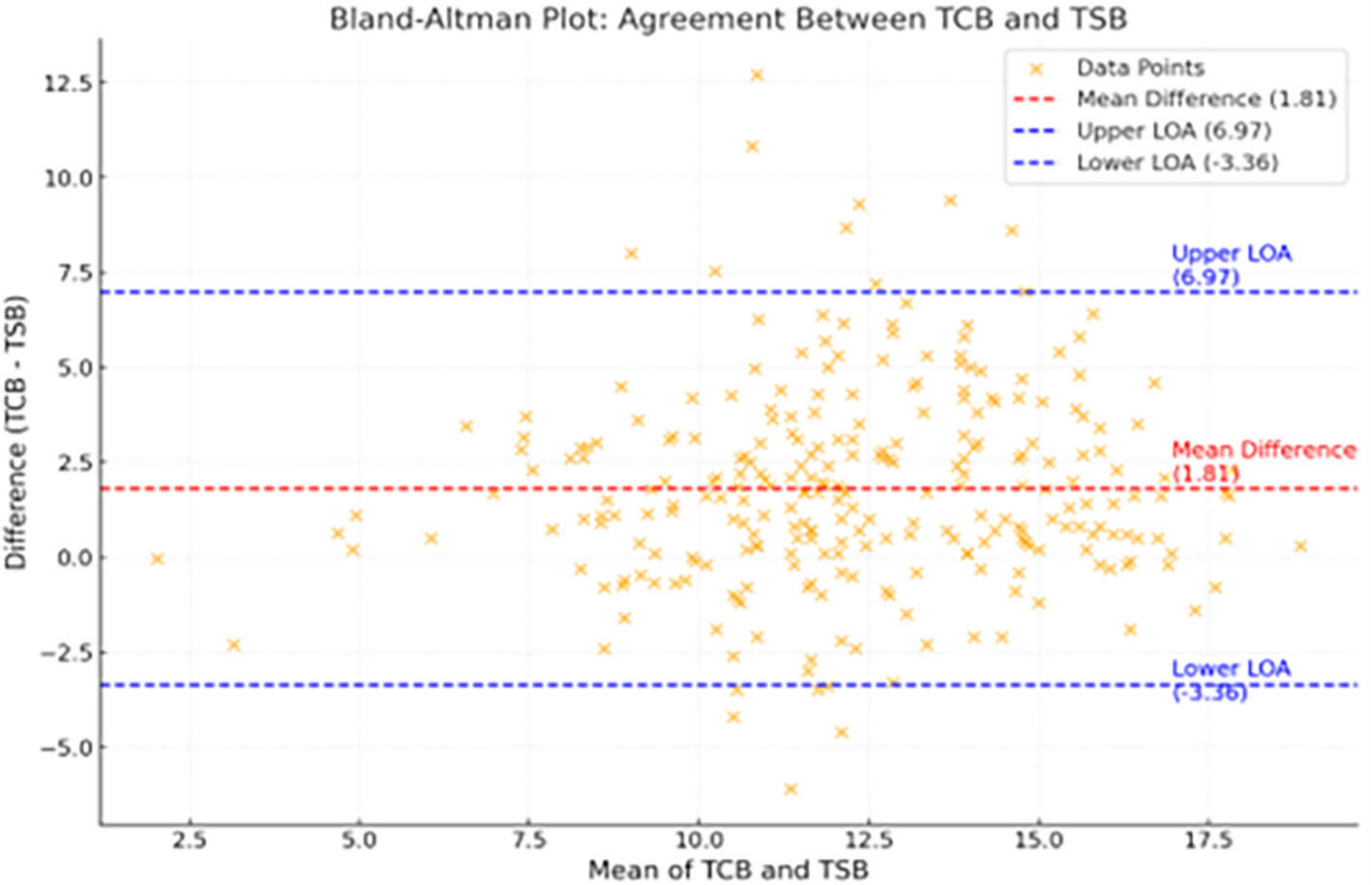
Bland Altman plot shows good agreement between TCB and TSB with a mean difference of 1.81mg/dl between the two values.

The Bland-Altman plot ( Figure 4) demonstrates good agreement between TCB and TSB levels, with a mean difference of **1.81 mg/dL** between the two values.

## Discussion

The use of TCB measurement is increasingly favoured in hospital postnatal wards and neonatal intensive care units (NICUs) due to its ability to provide early detection, prompt intervention, and timely treatment, ultimately reducing neonatal morbidity and mortality associated with neonatal jaundice. However, its widespread adoption remains limited, particularly in developing countries, due to cost concerns and limited data supporting its use. TCB offers a non-invasive, rapid alternative to TSB tests, reducing the need for painful blood draws. This study evaluated whether TCB measurements reliably correlate with TSB levels.

Our study found a strong positive correlation between TCB levels and TSB levels. A previous multicentric study by James A. et al. shows a correlation of 0.78 was observed, similar to the positive correlation found in our study. The mean difference between TCB and TSB was noted as 0.84 mg/dL, ^15^ while our findings also showed a close approximation between these two measurements. Surana et al. (2017) reported a strong correlation (r = 0.836) among 160 neonates of varying gestational ages, similar to our findings. ^7^

In the study by Arasar Seeralar et al., involving 267 neonates, there was a significant correlation between TCB and TSB levels, which aligns closely with our findings. ^8^ Majid Mansouri et al. ^14^ have also shown that TCB measurement is a reliable estimate of TSB levels in neonates. These studies support the use of TCB as an effective, non-invasive method for monitoring jaundice in newborns. The strong positive correlation between TCB and TSB levels with hours of life observed in this study is consistent with findings by Rahmawati D et al., a study conducted at Dr Soetomo General Hospital among neonates. ^9^

A high correlation between TCB and TSB has also been shown among infants of Asian descent, such as Indonesian, ^10^ Chinese, ^11^ Japanese, ^12^ and Myanmar. ^13^

In the Bland-Altman plot of this study, the bias line indicated the mean difference of 1.81 mg/dl between TCB and TSB. Most data points fell within ±1.96 times the SD of the difference between TSB and TcB values. This corroborates the strong agreement between TCB and TSB.

In resource-limited settings where the prevalence of prematurity is high, it often leads to prolonged NICU stays and phlebotomy-induced blood loss. Given the challenges of access to advanced laboratory techniques, TCB measurement is a more efficient and less invasive screening tool than visual assessment alone. It is quick, painless, and reliable for early identification of hyperbilirubinemia, reducing the reliance on invasive TSB tests. Regular TCB assessments can effectively guide early intervention, thereby improving neonatal outcomes.

The accuracy of TcB is known to be influenced by skin pigmentation, with several studies noting variations across different ethnic populations. ^15, 16^ South Indians predominantly belong to the Dravidian ethnic group and typically have darker skin tones compared to North Indian or Caucasian populations. Skin hydration is also another factor which can influence TcB measurements, although this has not yet been extensively studied and validated in literature. ^17^ Our center is in a coastal region where neonatal dehydration is relatively common. Accordingly, we included invasive serum bilirubin measurement as the reference standard for comparison, prioritizing patient safety and keeping in line with our institutional protocol.

While this may appear to increase invasive blood sampling, it provided robust paired data for correlation and Bland–Altman analysis, which is one of the best parameter for external validation. ^18^ Future multicentric studies in Indian settings should focus on validating TcB at recommended cut-offs, which would allow safe reduction of unnecessary blood sampling. Another limitation is the single-center design, which may limit generalizability, though our results provide important baseline evidence for South India.

## Conclusions

TCB estimation is a valuable non-invasive screening method for detecting neonatal hyperbilirubinemia. Its simplicity, rapid results, and ability to minimise painful blood sampling make it an excellent tool for monitoring jaundice in neonates, especially in settings with limited resources. This method can aid in timely identification and management, thus reducing morbidity associated with severe hyperbilirubinemia.

## Ethics and consent

This prospective study was carried out at a tertiary care NICU in a tier 2 city of south India following approval from the Institutional Ethics Committee, Kasturba medical college, Mangalore (Reg No. ECR/541/Inst/KA/2014/RR-20) with approval No IEC KMC MLR 08/2024/543 approved on 21/08/2024. The study is done as per STROBE guidelines for cross-sectional observational study. We adhered to all ethical parameters as per Declaraion of Helsinki. Written informed consent was taken from parents of newborns (mother or father).

## Data Availability

Figshare: CORRELATION OF TRANSCUTANEOUS AND SERUM BILIRUBIN LEVELS IN LATE PRETERM AND TERM NEONATES AT A TERTIARY CARE CENTER IN SOUTH INDIA. https://doi.org/10.6084/m9.figshare.28514285.v3. ^19^ The project contains the following underlying data:
1. TCB EXCEL sheet. xlsx 2. consent form of TCB study.docx Data are available under the terms of the Creative Commons Attribution 4.0 International license (CC-BY 4.0).
