# Correlation of transcutaneous and serum bilirubin levels in late preterm and term neonates at a tertiary care center in south India

**DOI:** 10.12688/f1000research.162608.3

**Published:** 2025-11-19

**Authors:** Doreswamy Chandranaik, Shahla Zakir, Laxmi Kamath, Nutan Kamath, Suchetha S Rao

**Affiliations:** 1 Department of Paediatrics, Kasturba Medical College Mangalore, Manipal Academy of Higher Education, Manipal, India

**Keywords:** Transcutaneous bilirubinometer; bilirubin; correlation analysis; phototherapy; Bland Altman analysis; noninvasive; Neonatal jaundice

## Abstract

**Background:**

Neonatal jaundice is one of the most prevalent conditions during first week of life causing morbidity and even mortality in few, especially in low – middle income countries. Although visual inspection for jaundice has been a time tested method, serum bilirubin is the gold standard investigation of choice. Due to this, newborns receive many heel or vein pricks for testing, hence the transcutaneous bilirubinometer can be a helpful non-invasive tool for diagnosing jaundice requiring phototherapy.

**Methods:**

This prospective study was carried out in a tertiary care hospital in Mangalore, Karnataka to compare a non invasive method of detecting bilirubin levels and serum bilirubin levels. Performance of a transcutaneous bilirubinometer Dräger Jaundice Meter JM-105 was assessed against routine venous serum bilirubin testing before phototherapy during neonatal care in the first two weeks of life. Results were derived by analysing the correlation coefficient between two methods and direct agreement was analysed using Bland Altman analysis.

**Results:**

Total of 271 neonates (>35 weeks) were included in the study. Transcutaneous bilirubinometry and serum bilirubin values were done on all of them in the first week of life. Correlation analysis showed significant relationship with a Pearson correlation coefficient of 0.629. Values of transcutaneous bilirubinometer showed excellent agreement with venous serum bilirubin concentration in Bland Altman analysis.

**Conclusions:**

The transcutaneous bilirubinometer is a reliable tool to screen neonates and identify those needing phototherapy there by reducing invasive blood sampling.

## Introduction

Neonatal hyperbilirubinemia is a prevalent condition in newborns, marked by the appearance of jaundice within the first week after birth. It affects approximately 60% of term neonates and up to 80% of preterm neonates. This condition occurs due to the accumulation of unconjugated bilirubin, a lipid-soluble pigment, in the skin and mucous membranes, leading to a yellowish discolouration. ^1^ Neonatal hyperbilirubinemia, while generally benign, is commonly seen postnatally in newborns. However, premature neonates and certain high-risk groups are more susceptible to severe forms, which, if not managed, can progress to complications like kernicterus. ^2^ Neonatal jaundice is clinically identified by a yellowish discolouration of the sclera, skin, and mucous membranes resulting from increased bilirubin levels in the bloodstream. The condition is categorised into two types: Unconjugated Hyperbilirubinemia and Conjugated Hyperbilirubinemia. ^3^ Phototherapy and exchange transfusion are the primary interventions for the prevention and management of bilirubin encephalopathy.

The primary methods for assessing bilirubin levels in newborns include visual inspection, measurement of total serum bilirubin, and transcutaneous bilirubinometry. Visual assessment is simple using Kramer’s rule but has notable limitations, as it is highly subjective; factors such as the physician’s experience, the baby’s skin colour, clothing, and lighting conditions can all influence the accuracy of visual estimation. Total serum bilirubin (TSB) measurement continues to be the gold standard for accurate assessment for monitoring bilirubin levels before and after phototherapy in both term and preterm neonates. However, obtaining blood samples via heel stick or venipuncture is not only painful and time-intensive but also elevates the risk of local and systemic infections, particularly in preterm neonates.

Transcutaneous bilirubin (TCB) assessment uses a handheld electronic device to measure bilirubin levels non-invasively on the skin’s surface, providing a painless and convenient method for screening jaundice in term and near-term neonates. The device, Transcutaneous Jaundice Detector (Drager Model MBJ20), utilises optical spectroscopy by emitting light into the skin and analysing the reflected wavelengths to estimate total serum bilirubin levels. It is increasingly accepted in clinical settings due to its simplicity and effectiveness. The National Institute for Health and Care Excellence (NICE) guidelines advise against using transcutaneous bilirubin (TCB) measurements within the first day of life or for neonates born before 35 weeks of gestation. Additionally, despite these limitations, TCB is a non-invasive screening method used to determine the need for phototherapy, and use of TCB can reduce infection risks. ^4^

In contrast, total serum bilirubin (TSB) measurement involves drawing a blood sample. The blood sample report is plotted on a nomogram, which is hour-specific to assess the neonatal hyperbilirubinemia risk. ^2^

Although TCB is an established screening method worldwide, its reliability may vary depending on ethnicity, skin pigmentation, hydration status and health system context. ^5, 6^ In India, available studies are relatively few ^7, 8^ and most are from limited sample sizes. This highlights a research gap, as findings from Western and East Asian populations ^9– 13^ cannot always be extrapolated to Indian neonates. Moreover, darker skin pigmentation may affect optical bilirubin detection, ^5^ underlining the need for region-specific validation. Against this backdrop, our study provides novel data by evaluating TcB in late preterm and term neonates in South India, with a larger sample size and additional analysis of correlation with hours of life and gestational age. This strengthens the evidence base for TcB adoption in resource-limited neonatal units especially southern India.

## Methods

This prospective study was carried out at a tertiary care NICU in a tier 2 city of south India following approval from the Institutional Ethics Committee, Kasturba medical college, Mangalore (Reg No. ECR/541/Inst/KA/2014/RR-20) with approval No IEC KMC MLR 08/2024/543 approved on 21/08/2024. The study is done as per STROBE guidelines for cross-sectional observational study. We adhered to all ethical parameters as per Declaration of Helsinki. The primary objective was to examine the correlation between transcutaneous bilirubin (TCB) and total serum bilirubin (TSB) levels in neonates with jaundice who required phototherapy.

The study included 271 neonates admitted to the NICU between August 2024 and December 2024. Both term and preterm neonates (>35 weeks) with clinical jaundice were included, while neonates with major congenital anomalies, skin conditions affecting the forehead or sternum, or those who had already received phototherapy or undergone exchange transfusions were excluded.

For each neonate, demographic details, antenatal history, maternal complications, feeding patterns, and clinical examination findings were documented using a structured pro forma. Bilirubin levels were assessed using two methods:

1. Transcutaneous Bilirubin (TCB): Measurements were taken using a Transcutaneous Jaundice Detector (Model MBJ20). Three readings were taken over the mid-sternum or forehead by the duty doctor, and their average was recorded in mg/dL.
2. Total Serum Bilirubin (TSB): Venous blood samples were collected, and TSB levels were measured using standard laboratory methods.

Each neonate underwent both TCB and TSB measurements within 15 minutes of each other and the paired values were analysed for correlation. Phototherapy was initiated for most neonates based on visual assessment, while a few were started on phototherapy later, guided by their TSB values. Data was systematically recorded in an Excel sheet.

### Sample size

To detect a mean difference of 0.23 mg/dL between TSB and TCB measurements with a statistical power of 80% and a significance level of p = 0.05, the required sample size was calculated as 271. This value considers the variability in measurements (σ=1.75) and the critical value for a 95% confidence interval (Zα/2=1.96} = 1.96). A design effect of 1.17 was incorporated to account for potential clustering or variability across different population subgroups. This adjustment ensures the study is adequately powered to detect clinically significant differences while maintaining the robustness of the results. This calculation aligns with findings from a previous study, which reported a mean TSB of 8.54 mg/dL and highlighted a standard deviation of 1.75 mg/dL in the average difference between TSB and TCB measurements, with negligible variation by gestational age or ethnicity. These findings provide a reliable basis for estimating the sample size required to achieve statistical validity in detecting the specified mean difference. ^14^

### Statistical analysis

Categorical variables were expressed as frequencies and percentages (%), while continuous variables were summarised as means ± standard deviations (SD) and medians with interquartile ranges (25th–75th percentiles). Pearson correlation coefficients were computed between TCB and TSB levels. Additional correlations were analysed separately for hours of life and gestational age to explore potential confounders. To visualise the stability of transcutaneous readings, the difference Δ = (TCB − TSB) was plotted against hours of life.

Pearson correlation coefficients were calculated between transcutaneous bilirubin (TCB) and total serum bilirubin (TSB) for the entire cohort and separately for two gestational groups-late preterm (35–36 weeks) and term (≥37 weeks) to assess consistency across maturities.

The difference Δ = (TCB − TSB) was plotted against gestational age to evaluate whether measurement bias varied with gestational age. A p < 0.05 was considered statistically significant. Bland-Altman analysis was performed to assess the agreement limits between TCB and TSB. Data entry was completed using Microsoft Excel, and statistical analyses were carried out using SPSS software (version 29).

## Results

A total of 271 neonates requiring phototherapy were included in the study to assess the correlation between transcutaneous bilirubin (TCB) and serum bilirubin (TSB) levels. Among the subjects, 140 (51.66%) were male neonates, and 131 (48.34%) were female neonates.

Table 1 provides a summary of the baseline characteristics of the study participants.

**Table 1.** Description of Baseline characteristics.

Baseline Characteristics	Value (n=271)
Mean gestational age (weeks)	38.34 ± 1.43
Mean hours of life at phototherapy (hours)	54.29 ± 27.2
Mean birth weight (kilograms)	2.91 ± 0.44
Male: Female	140:131 (1.06:1)
NVD (normal vaginal delivery)	49.07% (133)
LSCS (lower segment cesarean section)	50.9% (138)

**Figure 1.**
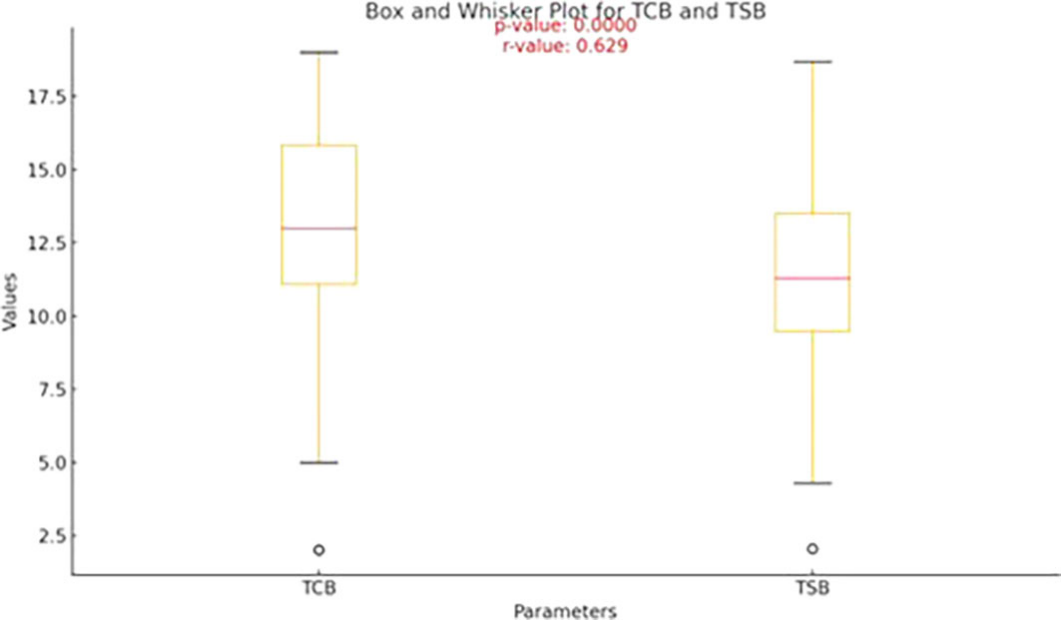
Box-and-whisker plot showing the median values of TCB and TSB.

Table 2 outlines the descriptive statistics for transcutaneous bilirubin (TCB) and serum bilirubin (TSB) levels ( Figure 1).

**Table 2.** Descriptive statistics of transcutaneous bilirubin and Serum Bilirubin.

Variable	Mean ± SD	Median (25th–75th percentile)	Range
Transcutaneous bilirubin (mg/dL)	13.2 ± 3.14	13 (11.1–15.85)	2–19
Serum bilirubin (mg/dL)	11.4 ± 2.97	11.3 (9.5–13.5)	2.04–18.7

**Figure 2.**
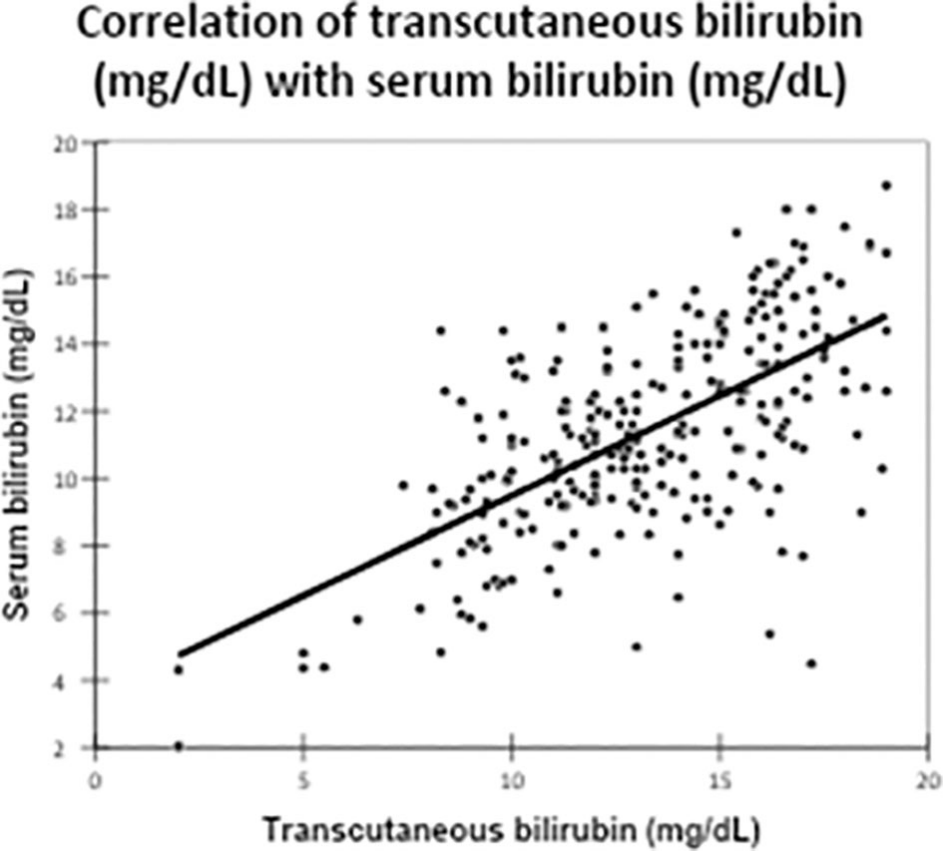
Correlation of transcutaneous bilirubin (mg/dL) with serum bilirubin (mg/dL).

There was a significant positive correlation between TCB and TSB levels, with a Pearson correlation coefficient of 0.629 ( Table 3, Figure 2). This correlation was statistically significant, with a p-value of <0.0001.

**Table 3.** Correlation of Transcutaneous bilirubin with serum bilirubin: Pearson correlation coefficient.

Variables	Serum bilirubin (mg/dL)	Transcutaneous bilirubin (mg/dL)
Pearson Correlation coefficient:		0.629
P value:		<0.0001

The correlation of TCB and TSB levels with gestational age is summarised in Table 4.

**Table 4.** Correlation of transcutaneous bilirubin and serum bilirubin with gestational age.

Group	Gestational range	n	Mean GA (weeks)	r (TCB vs TSB)	p value
Late preterm	35 – 36 + 6 weeks	42	35.8 ± 0.4	0.612	<0.001
Term	≥ 37 weeks	229	38.6 ± 0.8	0.633	<0.001

Pearson correlation coefficients were calculated between transcutaneous bilirubin (TCB) and total serum bilirubin (TSB) for the entire cohort and separately for two gestational group late preterm (35–36 weeks) and term (≥37 weeks) to assess consistency across maturities.

**Figure 3.**
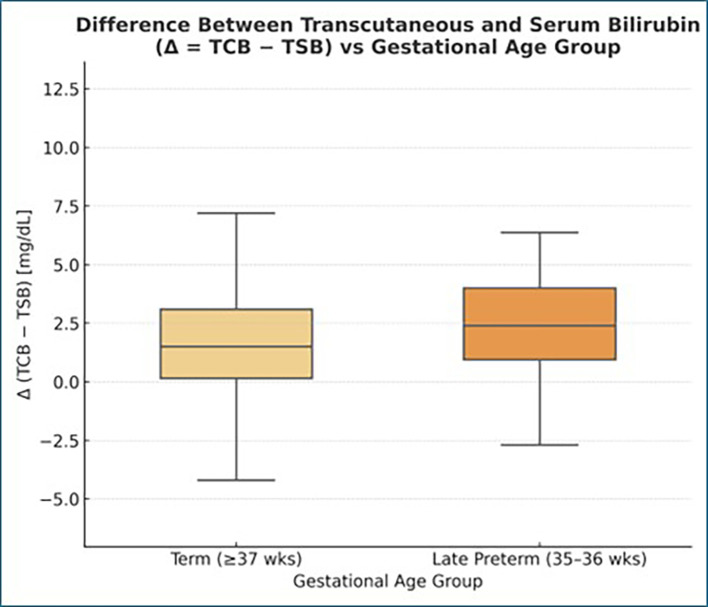
Correlation of gestational age with difference of transcutaneous bilirubin and ttotal serum bilirubin (mg/dL).

The difference Δ = (TCB − TSB) was plotted against gestational age ( Figure 3) to evaluate whether measurement bias varied with gestational age.

The difference between correlation coefficients was **statistically nonsignificant** (z = 0.19, p > 0.8), indicating that gestational maturity did not significantly alter the relationship between TCB and TSB. We also compared the difference **Δ = (TCB − TSB)** across gestational ages.

The bias remained consistent (+1.81 mg/dL on average) throughout 35–40 weeks, with **no visible trend** (r = −0.03, p > 0.05).

The mean difference (Δ = TCB − TSB) was **+1.84 mg/dL** in late-preterm infants (35–36 weeks) and **+1.79 mg/dL** in term infants (≥37 weeks), showing no significant difference between the two groups (p > 0.05).

**Figure 4.**
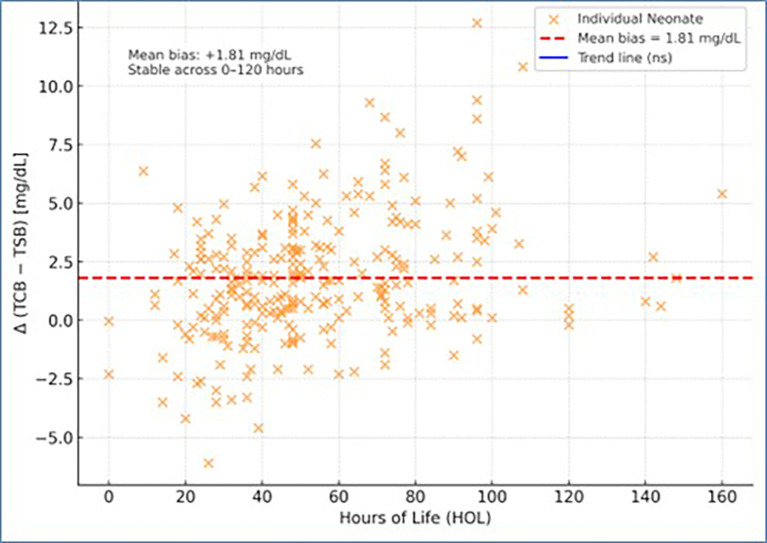
Correlation of hours of life with difference of transcutaneous bilirubin and total serum bilirubin.

The difference Δ = (TCB − TSB) plotted against hours of life showed that the mean bias of +1.81 mg/dL remained stable throughout the first five days of life (r = 0.08, p > 0.05). This indicates that transcutaneous bilirubin measurements maintained consistent agreement with serum values across the early neonatal period ( Figure 4).

**Figure 5.**
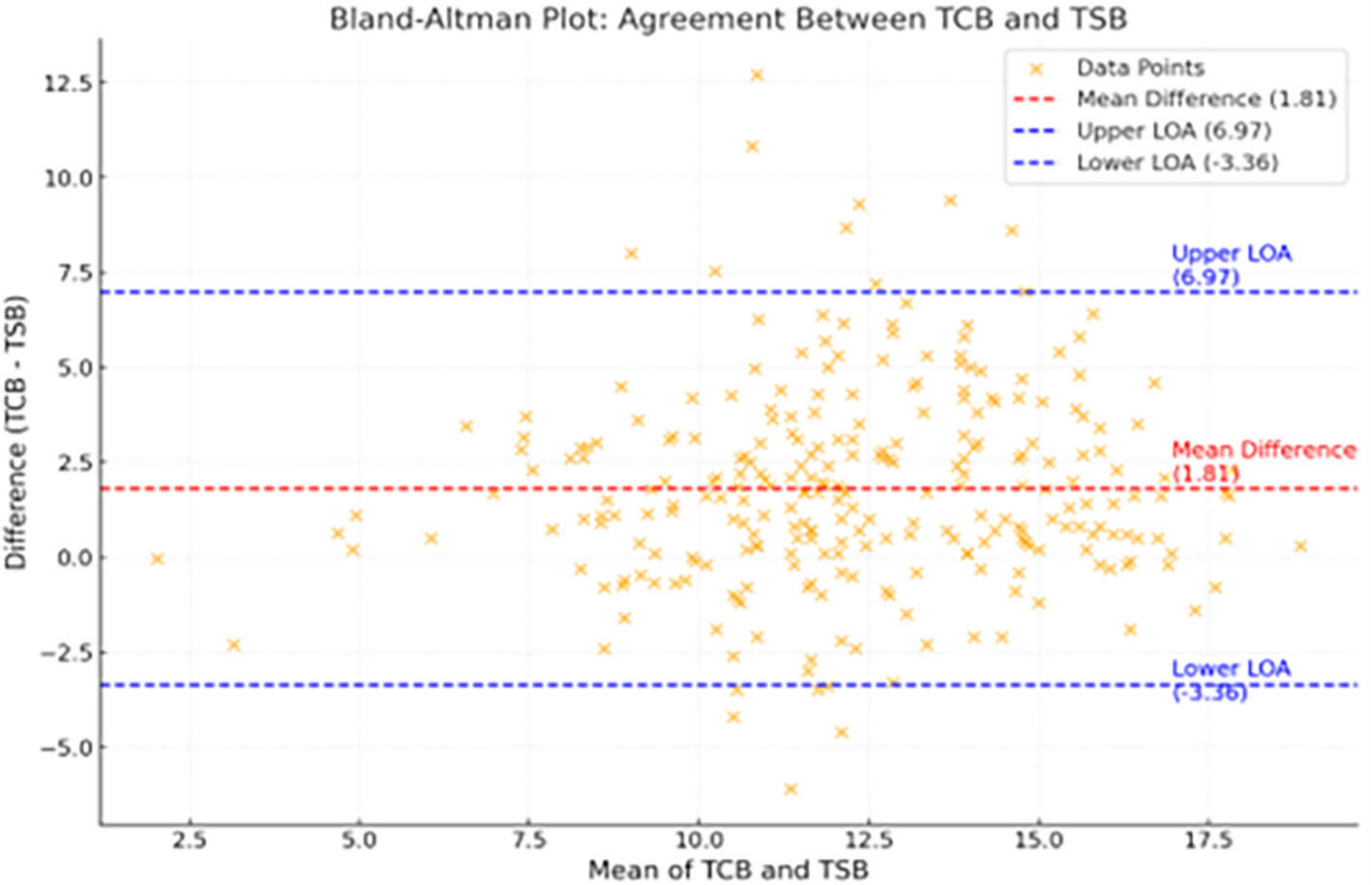
Bland Altman plot shows good agreement between TCB and TSB with a mean difference of 1.81 mg/dl between the two values.

The Bland-Altman plot ( Figure 5) demonstrates good agreement between TCB and TSB levels, with a mean difference of **1.81 mg/dL** between the two values.

## Discussion

The use of TCB measurement is increasingly favoured in hospital postnatal wards and neonatal intensive care units (NICUs) due to its ability to provide early detection, prompt intervention, and timely treatment, ultimately reducing neonatal morbidity and mortality associated with neonatal jaundice. However, its widespread adoption remains limited, particularly in developing countries, due to cost concerns and limited data supporting its use. TCB offers a non-invasive, rapid alternative to TSB tests, reducing the need for painful blood draws. This study evaluated whether TCB measurements reliably correlate with TSB levels.

Our study found a strong positive correlation between TCB levels and TSB levels. A previous multicentric study by Taylor et al. shows that a correlation of 0.78 was observed, similar to the positive correlation found in our study. The mean difference between TCB and TSB was noted as 0.84 mg/dL, ^15^ while our findings also showed a close approximation between these two measurements. Surana et al. (2017) reported a strong correlation (r = 0.836) among 160 neonates of varying gestational ages, similar to our findings. ^7^

In the study by Arasar Seeralar et al., involving 267 neonates, there was a significant correlation between TCB and TSB levels, which aligns closely with our findings. ^8^ Majid Mansouri et al. ^14^ have also shown that TCB measurement is a reliable estimate of TSB levels in neonates. These studies support the use of TCB as an effective, non-invasive method for monitoring jaundice in newborns. The strong positive correlation between TCB and TSB levels with hours of life observed in this study is consistent with findings by Rahmawati D et al., a study conducted at Dr Soetomo General Hospital among neonates. ^9^

A high correlation between TCB and TSB has also been shown among infants of Asian descent, such as Indonesian, ^10^ Chinese, ^11^ Japanese, ^12^ and Myanmar. ^13^

Plotting Δ (TCB − TSB) against hours of life showed no systematic trend, confirming stable performance of the device during the first five days of life. A few isolated outliers may represent infants with undiagnosed pathological jaundice or dehydration. Stratified analysis revealed that the agreement between TCB and TSB did not differ significantly between late-preterm and term neonates. The absence of systematic change in Δ (TCB − TSB) across 35–40 weeks suggests that gestational maturity does not materially affect the optical accuracy of transcutaneous bilirubinometry. Hence, TCB measurement can be reliably used for screening neonatal hyperbilirubinemia in infants ≥35 weeks in our population.

The Bland-Altman analysis in this study demonstrated a mean bias of +1.81 mg/dl, indicating that TcB values tended to be slightly higher than the corresponding TSB values. The 95% limits of agreement ranged from −3.36 mg/dl to +6.97 mg/dl, suggesting that, for most paired measurements, TcB values could be as much as 3.36 mg/dl lower or 6.97 mg/dl higher than TSB. Most data points fell within approximately 5.16 mg/dl of the mean bias, consistent with the calculated limits of agreement. Although the overall mean difference was small, the relatively wide limits indicate that individual discrepancies between TcB and TSB may be clinically significant, particularly at higher bilirubin levels.

Previous studies have demonstrated that a mean bias within ±2 mg/dL is considered acceptable for TCB-based screening, as confirmatory serum estimation is routinely performed when readings approach phototherapy thresholds. ^1^^,^^2^^,^^8^ Our observed mean bias of +1.81 mg/dL therefore falls well within this accepted range. Occasionally wider discrepancies between TCB and TSB measurements can be attributed to physiological and optical factors such as skin pigmentation, hydration status, and subcutaneous tissue thickness that influence light reflectance and absorption. Hence, the intended role of TCB is primarily as a **screening and triage device**, not a diagnostic substitute. A small positive bias is clinically desirable, as it promotes early identification and timely referral of neonates at risk for hyperbilirubinemia. ^5^^,^^8^^,^^10^

When we reviewed literature for other studies with different instruments we found that in a study done by Rubaltelli et al in Japan studied and compared two instruments viz BiliCheck and Minolta Airshields JM 102 with total serum bilirubin at different sites. The correlation coefficients ranged from 0.71 to 0.81, indicating good agreement between TCB and TSB levels. However like in our study they also had few outliers more in Minolta Airshields device. BiliCheck measures TcB by isolating bilirubin absorption from other factors such as hemoglobin and melanin using spectral subtraction. This allows TcB readings that are largely independent of race, gestational age, or birth weight, a finding supported by previous studies. ^16^

In another study done in Australia by Khajehei et al. JM-105 and MBJ-20 TcB measurements correlated strongly with each other and with serum bilirubin with correlation coefficient of 0.81, slightly overestimating SBR but providing reliable, high-sensitivity readings at lower levels and high-specificity readings at higher levels. In this study they found that chances of overestimating was equally high in both devices as they had relatively high rate of false positives. This interpretation was confirmed in the mean differences and limits of agreement plot which had outliers like in our study. This difference could be due to melanin content or exposed areas having continuous exposure to room light or natural light, which again explained why sternum area was more reliable than forehead. We had checked forehead and sternum area in our study. The few outliers in our study could be due to dark color of the skin in few babies, undiagnosed pathological jaundice, also hydration status of babies could contribute to overestimation in TCB. ^17^

In another study done by Madubuike et al used the MBJ-20 device to examine neonatal jaundice in 88 Nigerian neonates with gestational ages of 28 to 36 weeks. TcB measurements showed a strong positive linear relationship with TSB, with correlation coefficients of 0.904 for forehead readings and 0.917 for sternum readings, indicating excellent agreement across both sites. They also showed that compared with the forehead measurements, the sternum measurement using the MBJ-20 had greater correlation with the TSB in both preterm and term neonates and is a useful bilirubinometer for estimating TSB levels in neonates. The Bland altman plot analysis in this study although majority of the measurements fell within a 95% confidence interval, signifying strong agreement between the standard laboratory method and the MBJ20 transcutaneous bilirubinometer, also highlighted that the level of imprecision was great with an overestimation of bilirubin value like in our study. Clinical implication of such results is that TCB cannot replace TSB measurements in evaluation of severe jaundice and can only be used as surrogate marker. ^18^

The rate of cesarean deliveries in our cohort was relatively high compared to reports from Western populations. Our institution serves as a regional perinatal referral center, receiving mothers with complications such as preeclampsia, gestational diabetes, intrauterine growth restriction, and preterm labor, all of which contribute to an increased likelihood of operative delivery. Also, if we see the current obstetric trends in many tertiary-care centers in South India, cesarean delivery rates often range from 35–45%, particularly in referral hospitals that manage high-risk pregnancies. ^19^ Therefore, the higher cesarean rate observed in this study reflects the high risk obstetric population rather than a bias in sampling. Nevertheless, this should be considered when interpreting the generalizability of our findings to lower-risk or community settings thereby mandating more such studies from different cohorts of our country.

In resource-limited settings where the prevalence of prematurity is high, it often leads to prolonged NICU stays and phlebotomy-induced blood loss. Given the challenges of access to advanced laboratory techniques, TCB measurement is a more efficient and less invasive screening tool than visual assessment alone. It is quick, painless, and reliable for early identification of hyperbilirubinemia, reducing the reliance on invasive TSB tests. Regular TCB assessments can effectively guide early intervention, thereby improving neonatal outcomes.

The accuracy of TcB is known to be influenced by skin pigmentation, with several studies noting variations across different ethnic populations. ^15, 20^ South Indians predominantly belong to the Dravidian ethnic group and typically have darker skin tones compared to North Indian or Caucasian populations. Skin hydration is also another factor which can influence TcB measurements, although this has not yet been extensively studied and validated in literature. ^21^ Our center is in a coastal region where neonatal dehydration is relatively common. Accordingly, we included invasive serum bilirubin measurement as the reference standard for comparison, prioritizing patient safety and keeping in line with our institutional protocol.

While this may appear to increase invasive blood sampling, it provided robust paired data for correlation and Bland–Altman analysis, which is one of the best parameters for external validation. ^22^ Future multicentric studies in Indian settings should focus on validating TcB at recommended cut-offs, which would allow safe reduction of unnecessary blood sampling. Another limitation is the single-center design, which may limit generalizability, though our results provide important baseline evidence for South India.

## Conclusions

TCB estimation is a valuable non-invasive screening method for detecting neonatal hyperbilirubinemia. Its simplicity, rapid results, and ability to minimise painful blood sampling make it an excellent tool for monitoring jaundice in neonates, especially in settings with limited resources. This method can aid in timely identification and management, thus reducing morbidity associated with severe hyperbilirubinemia.

## Ethics and consent

This prospective study was carried out at a tertiary care NICU in a tier 2 city of south India following approval from the Institutional Ethics Committee, Kasturba medical college, Mangalore (Reg No. ECR/541/Inst/KA/2014/RR-20) with approval No IEC KMC MLR 08/2024/543 approved on 21/08/2024. The study is done as per STROBE guidelines for cross-sectional observational study. We adhered to all ethical parameters as per Declaraion of Helsinki. Written informed consent was taken from parents of newborns (mother or father).

## Data Availability

Figshare: CORRELATION OF TRANSCUTANEOUS AND SERUM BILIRUBIN LEVELS IN LATE PRETERM AND TERM NEONATES AT A TERTIARY CARE CENTER IN SOUTH INDIA. https://doi.org/10.6084/m9.figshare.28514285.v3. ^23^ The project contains the following underlying data:
1. TCB EXCEL sheet. xlsx 2. consent form of TCB study.docx Data are available under the terms of the Creative Commons Attribution 4.0 International license (CC-BY 4.0).
