# Supplementary material for: Correlation of transcutaneous and serum bilirubin levels in late preterm and term neonates at a tertiary care center in south India
Source: F1000Res. 2025 Nov 19;14:403. Originally published 2025 Apr 7. [Version 3] doi: 10.12688/f1000research.162608.3 (PMC12745854; doi:10.12688/f1000research.162608.3)
Supplement: Peer review history

# Reviewer report: Reviewer response for version 3

**DOI:** 10.5256/f1000research.191336.r434251

**Published:** 2025-12-28

**Authors:** Alyssa Shapiro

**Affiliations:** 1 The George Washington University, Washington, USA

The authors have made significant changes to the manuscript which I think make the results easy to interpret and the discussion very well written. I have a few minor additional comments, but I would say that once these are addressed, then this manuscript is ready for indexing.

  

1. Abstract –  The Abstract lists the brand of TCB device as Drager JM-105; could you please change this to Drager MBJ20 to reflect what is written in the body?
2. Introduction – Where you say “Additionally, despite these limitations,” I might instead just say “Despite these limitations,”
3. Introduction-  The intent of my previous comment on the Introduction was to rearrange the content in order to improve flow. You start with Jaundice in general, then talk about Visual Inspection and TSB, and then next go into detail about TCB, and where your study addresses a research gap with TCB. With that in mind, this second short paragraph about TSB could either be moved or deleted: “In contrast, total serum bilirubin (TSB) measurement involves drawing a blood sample… hyperbilirubinemia risk.”
4. Were all TCB and TSB pairs completed *before* phototherapy treatment was initiated? The Methods section states the criteria for starting phototherapy, but not the timing. Is possible that phototherapy treatment itself, started before TSB and/or TCB, could have an effects on the bilirubin readings by both methods. 
5. I personally disagree with this statement: “A small positive bias is clinically desirable, as it promotes early identification and timely referral of neonates at risk of hyperbilirubinemia.” I might instead make the following statement as my own opinion, “It is helpful for South Indian clinicians to be aware that the that the Drager MBJ20 TCB has a small positive bias in South Indian populations, because this gives clinicians helpful information to interpret TCB results accordingly when making decisions about individual patients.” However, I leave it up to the authors to leave their writing as is, or modify it if they would like, as I think the readers have the information needed to come to their own conclusions.

Is the work clearly and accurately presented and does it cite the current literature?

Yes

If applicable, is the statistical analysis and its interpretation appropriate?

Partly

Are all the source data underlying the results available to ensure full reproducibility?

Yes

Is the study design appropriate and is the work technically sound?

Yes

Are the conclusions drawn adequately supported by the results?

Partly

Are sufficient details of methods and analysis provided to allow replication by others?

Yes

Reviewer Expertise:

Bioengineering, Jaundice

I confirm that I have read this submission and believe that I have an appropriate level of expertise to confirm that it is of an acceptable scientific standard.

# Reviewer report: Reviewer response for version 2

**DOI:** 10.5256/f1000research.187250.r420926

**Published:** 2025-10-11

**Authors:** Alyssa Shapiro

**Affiliations:** 1 The George Washington University, Washington, USA

This article evaluated the accuracy of the Drager model MBJ20 transcutaneous bilirubin (TCB) compared to standard total serum bilirubin measurement in South India. This is an important research question, as TCB measurement provides a low-cost, noninvasive alternative to blood sampling, and South India - like many LMICs - are underrepresented in many research studies including those evaluating TCB accuracy. TCB performance is known to be population-dependent and can especially vary in darker skin tones. The authors mention that there are darker skin tones in South Indian populations along with frequent dehydration, both of which can affect TCB performance. The research methods presented here are well done. Below are some suggestions that can further improve the manuscript.

1. Major suggestions:
  1. In the Statistical Analysis section, what is meant by “accounting for hours of life and gestational age”? I believe that Figure 3 and Table 4 address hours of life, correct? I was confused by Figure 3, because the TCB increase in the first five days of life is known to vary greatly among individual newborns due to gestational age and risk factors. For example, if a newborn has pathological jaundice (for example, due to hemolytic anemia) rather than physiological jaundice, the rate of bilirubin increase is much higher. I think that if you are looking to show the utility of the TCB throughout the first five days of life, one possibility could be to show “TCB-TSB” on the Y axis, compared to “Hours of life” on the X axis.  
  2. Likewise, in Table 4, I found it confusing to do a direct correlation of TCB to gestational age.  I would recommend instead doing 1) Pearson correlation coefficient of TCB and TSB, for two groups, one of term newborns and one of preterm newborns, then compare those two correlation coefficients, or 2) Compare “TCB – TSB” vs gestational age (perhaps a graph like Figure 2), and show that this difference does not change drastically for different gestational ages.
  3. I see that your Result is that there is a mean bias of +1.81 mg/dL, and the 95% limits of agreement are -3.36 – 6.97. Your Conclusion is that the there is good agreement between TCB and TSB levels. I personally disagree with your Conclusion; or rather, to me it’s a bit concerning that there is this mean bias and also that many individual data points show a huge difference between TCB and TSB (for example, TCB of around 16 when TSB is around 5). However, your results do align quite well with other studies that evaluate TCB vs. TSB, so I’m not doubting the accuracy of your results, only your interpretation of them. I would suggest adding some text in the Discussion along those lines. Or rather, could you please further elaborate about why you conclude that the agreement is good?
  4. Related to the previous statement, I would suggest commenting further on how your results compare to other studies evaluating both TCB in general, as well as the Drager model MBJ20 in particular. References 7-15 are a good start; what were their Pearson’s values? Which brand of TCB device did they use? Did they also have some measurements with such a large difference between TSB and TCB? Can you include some other references that specifically evaluate the MBJ20?
  5. Could you please comment on why some individual measurements may have such a discrepancy (for example, TCB around 16 when TSB is around 5)? Did these newborns have particular pre-existing conditions? Were they preterm newborns?
  6. Do you have some theories as to why you found a positive overestimation (+1.81 mg/dL) in your study? Do you think it’s related to the darker skin tones of your population? If so, could you comment further on this in the Discussion?
2. Minor suggestions:
  1. Could you please briefly clarify that all measurements were taken before phototherapy began? Also, how was it determined that neonates required phototherapy? (Via visual inspection?)
  2. Most data points fell within +/- 1.96 times the SD of the difference between TSB and TcB values  --- I would remove this statement, as by definition, 95% data points on a Bland-Altman plot will fall within +/- 1.96 SD. I would instead modify this statement to say something like, ‘Most data points fell within 5.16 mg/dL of the mean bias’.
  3. Could you be more specific about how much time had passed between TCB and TSB measurements? You say ‘at the same time’; could you instead say something like ‘within 30 minutes of each other,’ ‘within 15 minutes of each other,’ etc?
  4. The C-section rate is quite high. Could you please comment on whether this is an expected finding? Perhaps this is normal in south India, but American authors might be surprised and curious about this. If this reflect that this hospital sees a higher-risk population than the general population, that might affect the generalizability of your findings (which you have already commented on in the Discussion).
3. Minor suggestions to improve the English writing: (Overall the English & writing were good):

Abstract:

- One of the most prevalent condition --> one of the most prevalent conditions
- Due to this newborn receive many heel or vein pricks for testing, hence transcutaneous bilirubinometer can --> Due to this, newborns receive many heel or vein pricks for testing, hence the transcutaneous bilirubinometer can

Introduction:

- “The primary methods for assessing bilirubin levels in newborns include visual inspection, transcutaneous bilirubinometry, and measurement of total serum bilirubin. Visual assessment is simple using Kramer’s rule but has notable limitations, as it is highly subjective; factors such as the physician's experience, the baby's skin colour, clothing, and lighting conditions can all influence the accuracy of visual estimation. Transcutaneous bilirubinometry provides a non-invasive alternative, whereas total serum bilirubin measurement continues to be the gold standard for accurate assessment. Requiring a blood sample for confirmation, especially in high-risk cases. Transcutaneous bilirubin (TCB) assessment uses a handheld electronic device to measure bilirubin levels non-invasively on the skin's surface, providing a painless and convenient method for screening jaundice in term and near-term neonates. It is increasingly accepted in clinical settings due to its simplicity and effectiveness. The device, Transcutaneous Jaundice Detector (Drager Model MBJ20). utilises optical spectroscopy by emitting light into the skin and analysing the reflected wavelengths to estimate total serum bilirubin levels. This method offers a reliable alternative for early jaundice detection without requiring blood draws. ^4^”

--> “The primary methods for assessing bilirubin levels in newborns include visual inspection, measurement of total serum bilirubin, and transcutaneous bilirubinometry. Visual assessment is simple using Kramer’s rule but has notable limitations, as it is highly subjective; factors such as the physician's experience, the baby's skin colour, clothing, and lighting conditions can all influence the accuracy of visual estimation. Total serum bilirubin (TSB) measurement continues to be the gold standard for accurate assessment for monitoring bilirubin levels before and after phototherapy in both term and preterm neonates. However, obtaining blood samples via heel stick or venipuncture is not only painful and time-intensive but also elevates the risk of local and systemic infections, particularly in preterm neonates.

 Transcutaneous bilirubin (TCB) assessment uses a handheld electronic device to measure bilirubin levels non-invasively on the skin's surface, providing a painless and convenient method for screening jaundice in term and near-term neonates. The device, Transcutaneous Jaundice Detector (Drager Model MBJ20). utilises optical spectroscopy by emitting light into the skin and analysing the reflected wavelengths to estimate total serum bilirubin levels. It is increasingly accepted in clinical settings due to its simplicity and effectiveness. The National Institute for Health and Care Excellence (NICE) guidelines advise against using transcutaneous bilirubin (TCB) measurements within the first day of life or for neonates born before 35 weeks of

 gestation. Additionally, Despite these limitations, TCB is a non-invasive screening method used to determine the need for phototherapy, and use of TCB can reduce infection risks.

 ^This is one quick possibility of rearranging the Introduction to reduce repetitive sentences and incomplete sentences, as well as to keep all the TSB info together and TCB info together.

 Sample Size:

- A design effect 1.17 --> A design effect of 1.17

Discussion:

-  A previous multicentric study by James A. et al. shows a correlation of 0.78 was observed, similar to the positive correlation found in our study. --> A previous multicentric study by Taylor et al. shows that a correlation of 0.78 was observed, similar to the positive correlation found in our study.
- Which is one of the best parameter for external validation --> Which is one of the best parameters for external validation

Is the work clearly and accurately presented and does it cite the current literature?

Yes

If applicable, is the statistical analysis and its interpretation appropriate?

Partly

Are all the source data underlying the results available to ensure full reproducibility?

Yes

Is the study design appropriate and is the work technically sound?

Yes

Are the conclusions drawn adequately supported by the results?

Partly

Are sufficient details of methods and analysis provided to allow replication by others?

Yes

Reviewer Expertise:

Bioengineering, Jaundice

I confirm that I have read this submission and believe that I have an appropriate level of expertise to confirm that it is of an acceptable scientific standard, however I have significant reservations, as outlined above.

# Reviewer report: Reviewer response for version 2

**DOI:** 10.5256/f1000research.187250.r411195

**Published:** 2025-09-19

**Authors:** Sanjoy Kumer Dey

**Affiliations:** 1 Bangabandhu Sheikh Mujib Medical University, Dhaka, Bangladesh

Thanks for allowing me to review again.

 I’ve gone through it

 it seems all comments have been addressed

Is the work clearly and accurately presented and does it cite the current literature?

Partly

If applicable, is the statistical analysis and its interpretation appropriate?

Yes

Are all the source data underlying the results available to ensure full reproducibility?

Yes

Is the study design appropriate and is the work technically sound?

Yes

Are the conclusions drawn adequately supported by the results?

Yes

Are sufficient details of methods and analysis provided to allow replication by others?

Yes

Reviewer Expertise:

neonatal health

I confirm that I have read this submission and believe that I have an appropriate level of expertise to confirm that it is of an acceptable scientific standard.

# Reviewer report: Reviewer response for version 1

**DOI:** 10.5256/f1000research.178836.r395736

**Published:** 2025-08-22

**Authors:** Sanjoy Kumer Dey

**Affiliations:** 1 Bangabandhu Sheikh Mujib Medical University, Dhaka, Bangladesh

It is already established method to detect Neonatal Jaundice by doing TcB. In introduction author could search more literatures to find the research gap. Rationale of the study is not clearly mentioned. It needs to be clear what new information is generated and novelty of the study which seems missing in this article.

 From ethical point of view as it is established that there are some cut off values determined by TcB upon which TSB is recommended to send to avoid unnecessary blood sampling but here it is being observed that all babies having jaundice detected by TcB undergone blood sampling. Alternatively they could compare TcB value where TSB is recommended to prove it to be sound ethically.

Is the work clearly and accurately presented and does it cite the current literature?

Partly

If applicable, is the statistical analysis and its interpretation appropriate?

Yes

Are all the source data underlying the results available to ensure full reproducibility?

Yes

Is the study design appropriate and is the work technically sound?

Yes

Are the conclusions drawn adequately supported by the results?

Yes

Are sufficient details of methods and analysis provided to allow replication by others?

Yes

Reviewer Expertise:

neonatal health

I confirm that I have read this submission and believe that I have an appropriate level of expertise to confirm that it is of an acceptable scientific standard, however I have significant reservations, as outlined above.

# Response

**Published:** 2025-08-25

**Authors:** laxmi kamath

**Affiliations:** Pediatrics, Kasturba medical college Mangalore, Manipal Academy of Higher Education, Karnataka, Manipal,576104, India, India

**Author Response to Reviewer Comments**

 We sincerely thank the reviewer for the thoughtful and constructive feedback. Below we provide a point-by-point response, and have revised the manuscript (Version 2) accordingly.

 Comment 1:

*“It is already established method to detect Neonatal Jaundice by doing TcB. In introduction author could search more literatures to find the research gap. Rationale of the study is not clearly mentioned. It needs to be clear what new information is generated and novelty of the study which seems missing in this article.”*

**Response:**

  We agree with the reviewer. While TcB is indeed an established screening tool, its performance may vary by ethnicity, skin pigmentation, hydration status and healthcare context. Data from India remain relatively limited, with only a few published studies¹-², often with smaller sample sizes. By contrast, most validation studies have been conducted in Western or East Asian populations³⁻⁷, which cannot always be extrapolated to the Indian subcontinent.

 To address this, we have added a new paragraph in the Introduction (Version 2) highlighting the research gap and rationale. The novelty of our work lies in:

- Providing one of the larger single-center datasets from South India (n=271) evaluating TcB against TSB.
- Assessing TcB correlation not only with TSB but also with hours of life and gestational age, which adds clinically relevant insight.
- Generating population-specific evidence in darker-skinned neonates, thereby strengthening the case for wider adoption of TcB in Indian neonatal practice.

**Comment 2:**

*“From ethical point of view as it is established that there are some cut off values determined by TcB upon which TSB is recommended to send to avoid unnecessary blood sampling but here it is being observed that all babies having jaundice detected by TcB undergone blood sampling. Alternatively they could compare TcB value where TSB is recommended to prove it to be sound ethically.”*

**Response:**

  We acknowledge this important ethical concern. International guidelines suggest using TcB thresholds to decide when TSB is required⁸⁻⁹. However, at the time of our study TcB was not validated in our population and was not part of routine NICU practice.

 The accuracy of TcB is known to be influenced by skin pigmentation, with several studies noting variations across different ethnic populations. ^10,11^ Since South Indians predominantly belong to the Dravidian ethnic group and typically have darker skin tones compared to North Indian or Caucasian populations, we aimed to evaluate the reliability of TcB in this group.  Skin hydration is also another factor which can influence TcB measurements, although this has not yet been extensively studied and validated in the literature. ^12,13^ Our center is in a coastal region where neonatal dehydration is relatively common. Accordingly, we included invasive serum bilirubin measurement as the reference standard for comparison, prioritizing patient safety and keeping in line with our institutional protocol. This allowed us to generate paired TcB–TSB data for direct correlation and Bland–Altman analysis, which is one of the best parameter for external validation. ^14^

 We agree that a future step would be to validate TcB specifically at recommended cut-offs in Indian neonates, which would reduce unnecessary blood sampling. To clarify this, we have added a new paragraph in the Discussion (Version 2) explicitly addressing this ethical aspect and acknowledging the limitation.

 The findings from our study may offer valuable insights for pediatricians and neonatologists working in similar coastal environments and resource-limited settings.

**Comment 3:**

*“Is the work clearly and accurately presented and does it cite the current literature? – Partly.”*

**Response:**

  We have now strengthened the Introduction and Discussion with additional references already cited in our paper¹⁻⁷ to better contextualized our findings and support the novelty. This addresses the reviewer’s concern and ensures alignment with existing literature.

**References: **

1. Surana AU, Patel S, Prasad R, et al. Comparison of transcutaneous bilirubin with serum bilirubin measurements in neonates at a tertiary care center in the western part of India. *Int J Contemp Pediatr*. 2017;4:1445–3283.
2. Arasar Seeralar AT, Ganesh J, Suganya M, et al. Correlation between transcutaneous and serum bilirubin measurements in neonates in a tertiary neonatal care center. *Int J Contemp Med Res*. 2016;10(8):272–274.
3. Rahmawati D, Sampurna MTA, Etika R, et al. Transcutaneous bilirubin level to predict hyperbilirubinemia in preterm neonates. *F1000Res*. 2020;9:300.
4. Rohsiswatmo R, Oswari H, Amandito R, et al. Agreement test of transcutaneous bilirubin and bilistick with serum bilirubin in preterm infants receiving phototherapy. *BMC Pediatr*. 2018;18:315.
5. Ho EY, Lee SY, Chow CB, et al. BiliCheck transcutaneous bilirubinometer: a screening tool for neonatal jaundice in the Chinese population. *Hong Kong Med J*. 2006;12(2):99–102.
6. Yamana K, Morioka I, Kurokawa D, et al. Evaluation of BiliCare transcutaneous bilirubin device in Japanese newborns. *Pediatr Int*. 2017;59:1058–1063.
7. Yasuda S, Suzuki H, Htun Y, et al. Hour-specific nomogram for transcutaneous bilirubin in newborns in Myanmar. *Pediatr Int*. 2020;62:1049–1053.
8. Sharma AK, Dhawan K, Makkar M, et al. A correlation study between transcutaneous bilirubin and total serum bilirubin levels among neonates. *Asian J Pharm Clin Res*. 2022;10(3):272–274.
9. Raba AA, O'Sullivan A, Miletin J. Transcutaneous bilirubinometry during and after phototherapy in preterm infants: a prospective observational study. *BMJ Paediatr Open*. 2020;4(1):e000681.
10. Dam-Vervloet AJ, Morsink CF, Krommendijk ME, Nijholt IM, van Straaten HL, Poot L, Bosschaart N. Skin color influences transcutaneous bilirubin measurements: a systematic in vitro evaluation. Pediatric Research. 2025 Apr;97(5):1706-10.
11. Maya-Enero S, Candel-Pau J, Garcia-Garcia J, Duran-Jordà X, López-Vílchez MÁ. Reliability of transcutaneous bilirubin determination based on skin color determined by a neonatal skin color scale of our own. European Journal of Pediatrics. 2021 Feb;180(2):607-16.
12. van Erk MD, Dam-Vervloet AJ, de Boer FA, Boomsma MF, Straaten HV, Bosschaart N. How skin anatomy influences transcutaneous bilirubin determinations: an in vitro evaluation. Pediatric research. 2019 Oct;86(4):471-7.
13. Ercan Ş, Özgün G. The accuracy of transcutaneous bilirubinometer measurements to identify the hyperbilirubinemia in outpatient newborn population. Clinical biochemistry. 2018 May 1;55:69-74.
14. Krobath DM, Naumova EN, Cuevas AG, Sacheck JM, Wilson NL, Economos CD. Use of Bland-Altman Analysis to Examine the Racial and Ethnic Representativeness of Study Populations in Community-Based Pediatric Health Research. JAMA network open. 2023 May 1;6(5):e2312920-.
